# Social Networks in Dementia

**DOI:** 10.1093/geroni/igaf122.1164

**Published:** 2025-12-31

**Authors:** Sara Moorman, Alyssa Goldman, Hui Liu

## Abstract

Dementia poses significant barriers to the maintenance of social network relationships while also introducing new forms of social support needs from network members. These papers examine social network characteristics of older adults living with and without dementia. Patterson examines family networks, finding that people with dementia are as likely as people without to receive care from partners and siblings, but are more likely to receive care from adult children and grandchildren. Lin et al. investigate family care networks during COVID-19, also finding that people with dementia are more likely to receive care from adult children, although rates of filial care differ by race/ethnicity, age, and education. McConnell and Haggar measure the social networks of primary dementia caregivers, finding that larger caregiver networks are associated with greater primary caregiver stress, while greater network coordination is a source of caregiver support. Moorman and Goldman focus on the last five years of life, finding that discussion network size declines in the years before death among decedents without dementia but remains smaller and stable among decedents with dementia. Torres et al. use the Health and Retirement Survey linked with Medicare data to find that socially isolated older adults are more likely to experience Medicare-based under-diagnosis of dementia when compared with survey-based measures of cognitive status. These papers highlight the importance of social network relationships in dementia care, and collectively underscore the need to prioritize familial and community-based support systems prior to dementia onset in the wake of projected increases in persons living with dementia.

